# Comparison of deep and conventional machine learning methods in predicting joint moments in patients with cerebral palsy

**DOI:** 10.1007/s11517-026-03548-6

**Published:** 2026-03-17

**Authors:** Mustafa Erkam Özates, Firooz Salami, Sebastian Immanuel Wolf, Yunus Ziya Arslan

**Affiliations:** 1 Present Address: Electrical Electronics Engineering, Faculty of Engineering, Turkish- German University, Merkez, Merkez, Şahinkaya Cd. No:106, 34820, 34820 Beykoz, İstanbul, Türkiye; 2 Present Address: Clinic for Orthopaedics and Trauma Surgery, Heidelberg University Hospital, Schlierbacher Landstraße 200a, 69118 Heidelberg, Germany; 3 Department of Robotics and Intelligent Systems, The Institute of the Graduate Studies in Science and Engineering, Turkish-German University, Merkez, Şahinkaya Cd. No:106, 34820, 34820 Beykoz, İstanbul, Türkiye

**Keywords:** Gait analysis, Cerebral palsy, Machine learning, Joint moments

## Abstract

The hip, knee, and ankle joint moments during gait are critical for clinical decision-making in patients with cerebral palsy (CP). These moments are typically calculated using inverse dynamics and human body models based on ground reaction forces (GRF). However, obtaining GRF data from CP patients can be challenging. Recent studies suggest joint moments in CP patients can be predicted using joint angles alone, bypassing the need for GRF, through machine learning (ML). However, the optimal type and scope of input data for such models remain unclear. This study aimed to identify the most feasible ML approach based on prediction accuracy for predicting joint moments from gait kinematics in CP patients. Retrospective gait data from 917 CP patients were analysed; after applying inclusion-exclusion criteria, data from 622 CP patients were used. We evaluated four conventional ML algorithms, ridge regression, k-nearest neighbors, random forest, and multilayer neural network, using feature-based input, and two deep learning algorithms, one-dimensional convolutional neural network and long short-term memory network, using raw data input, with each model’s hyper-parameters optimized in a problem-specific manner. Models were assessed using normalized root mean square error (nRMSE) and Pearson correlation coefficient (PCC). Deep learning models achieved an average nRMSE of 14.75 ± 7.10 and PCC of 0.95, while conventional ML models yielded 16.03 ± 6.55 and 0.94, indicating both conventional and deep learning approaches showed promise for predicting joint moments in patients with CP. By considering factors such as data availability and computational cost, an appropriate ML method can be selected to effectively address gait kinetics prediction in individuals with CP.

**Graphical abstract:**

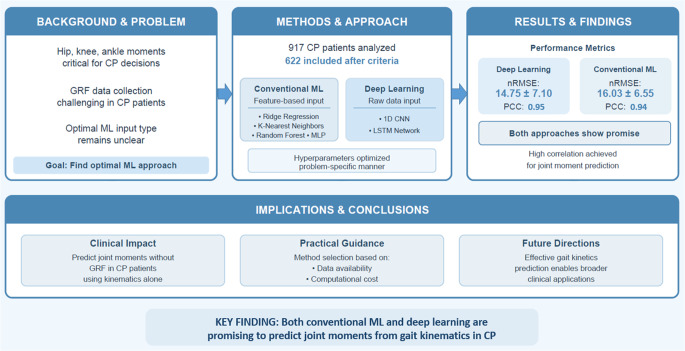

## Introduction

Cerebral Palsy (CP) affects neuromotor functions, and the lower limb joint moments are crucial for CP assessment, monitoring, and treatment [1–5]. Joint moments offer insights into muscle behaviors during joint motion. Obtaining joint moments in clinical gait analysis is challenging because it requires ground reaction force (GRF) measurements to be incorporated into inverse dynamics and human body models [6, 7]. Measuring GRF during natural walking is difficult and even more problematic for CP patients with deviated gaits [8, 9].

To address these challenges, researchers have explored predicting joint moments using machine learning (ML) techniques or musculoskeletal-based predictive models [10–15]. ML has proven to be a powerful tool in handling the issues posed by missing measurements and the lack of complete physical models. However, most efforts have focused on ML-based predictions of joint moments in typically developed (TD) individuals during gait. For example, researchers have successfully used feed-forward and long short-term memory neural networks to predict joint moments in TD subjects, utilizing kinematic data obtained from three-dimensional motion capture [16]. Additionally, a study employed a wavelet neural network that incorporated frequency information to effectively predict joint moments in TD subjects using both kinematic and electromyography data [17]. In our previous study [18], we predicted the lower limb joint moments based on the trunk, pelvis, hip, knee, and ankle kinematics during gait in patients with CP using a one-dimensional convolutional neural network. We found that ML based prediction of joint moments using kinematic data could be an alternative technique to conventional joint moment calculation in the gait analysis of patients with CP.

The data representation approaches and ML algorithms in the context of predicting gait kinetics from gait kinematics were not extensively explored in the studies mentioned above. When problem-specific features are carefully selected, the manual extraction of the features from raw data can simplify the learning process for the applied ML algorithms. In contrast, deep learning algorithms have the capability to automatically extract features, enabling them to explore a broader solution space.

A comprehensive analysis involving feature-based and raw data-based inputs, along with various conventional and deep ML algorithms, would enable us to evaluate and compare the predictive capabilities of different input variables and ML techniques. Accordingly, we aimed to compare the performance of different input and algorithm settings for predicting dorsiflexion-plantarflexion, knee flexion-extension, hip flexion-extension, and hip adduction-abduction moments in this study. The input sets comprised two types of data, one consisting of the manually extracted well-accepted features and the other consisting of the measured joint angles themselves. By exploring various combinations of input data and ML algorithms, we aimed to identify the most feasible approach, based on prediction accuracy, for estimating joint moments in patients with CP during gait.

## Materials and methods

### Subjects

The local ethical committee of the Heidelberg University Hospital approved the study (S-227/2021). Anonymized retrospective gait data from 917 CP patients were used. The CP patients had spastic diplegia with Gross Motor Function Classification System (GMFCS) levels I and II. No age or gender criteria were set for inclusion.

**Fig. 1:**
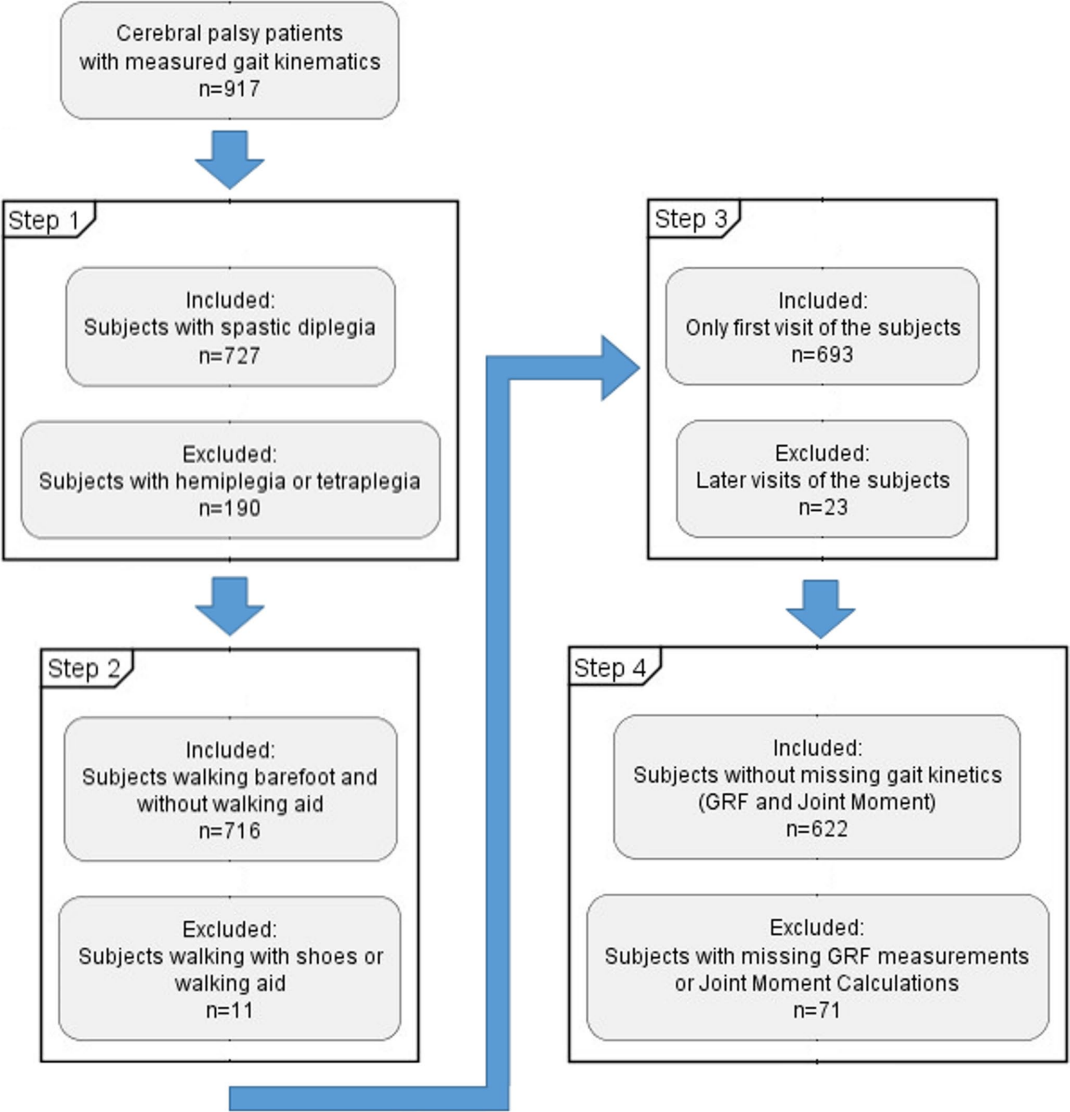
Inclusion-exclusion flow of the subjects with cerebral palsy. GRF: Ground reaction force

Kinematic data were collected from subjects using the Plugin Gait Model (Oxford Metrics, Oxford, UK) with a 19-marker setup. A 12-camera motion capture system (Vicon Motion Systems Ltd., Oxfordshire, UK) recorded the data as subjects walked at their self-selected speed. GRF data were simultaneously captured using two force plates (Kistler Instruments, Winterthur, Switzerland). Joint moments, normalized to body mass, were computed using an inverse kinematics model [19]. The first visits of patients with CP presenting spastic diplegia who could walk barefoot without assistive devices and with no missing measurements were included (Fig. 1).

### Datasets

The dataset includes kinematic data from the body parts (trunk, pelvis, hip, knee, and ankle) measured in three planes (sagittal, coronal, and transverse), making a total of 15 angles. Additionally, kinetic data for flexion-extension moments of the ankle, knee, and hip, as well as the adduction-abduction moment of the hip, were considered.

To process the data, all measurements were averaged across 7–10 strides for each subject and then normalized to a percentage of the gait cycle. The choice of averaging across 7–10 strides reflects subject-specific variability in the number of valid trials and ensures a consistent and representative gait pattern for each participant. Each resulting time series consisted of 101 points corresponding to 0–100% of the gait cycle. The dataset also contains the standard deviations of the time series throughout the strides for further analysis.

We focused on constructing different input sets that encompassed the same range of kinematic information. Specifically, two sets of inputs.

i) one for deep learning including raw kinematic time series.
ii) one for conventional ML algorithms that is composed of manually extracted features were constructed to predict joint moments.

These two distinct types of input sets were created using the collected kinematic data. The first type of input set comprised solely the stance phase of the raw kinematic time series themselves. The second type of input set involved stacking the extracted features from the kinematic time series, which were obtained following the Automated Feature Assessment Workflow for Instrumented Gait Analysis developed by Wolf et al. [20]. These extracted features captured relevant information from the time series to be used in conventional ML algorithms.

#### The first type of dataset for the deep learning

While gait-cycle normalization resulted in 101 time points (0–100%), only the stance phase was retained and temporally standardized to 60 time points for model input to ensure uniformity in the data size for training the ML algorithms. Since each subject may have a different duration of stance time, a standard interpolation technique was employed to adjust the time series to this standardized length. This interpolation process allowed for consistent data size across all subjects, facilitating the ML training process.

Furthermore, to ensure fair and unbiased learning, all time-series values, regardless of their unit or magnitude, were normalized to a common range between 0 and 1. This normalization technique prevented any particular time series with higher magnitudes from dominating the learning process. By scaling the values within a standardized range, the ML algorithms could effectively analyze and compare the patterns and relationships within the data.

Following the normalization step, the 15 kinematic time series, along with their corresponding standard deviations, were organized and stacked into a matrix format. This matrix, namely the input matrix, had 30 rows, representing 15 time-series and their associated standard deviations, and 60 columns, corresponding to the standardized length of the stance phases. This matrix format allowed for a structured and consistent representation of the data.

Each 30 × 60 matrix represents a single subject-specific input instance, resulting in a total of 622 matrices used for training and evaluation. These input matrices enabled the learning and prediction of joint moments based on the kinematic information and were used as the first type of dataset for training and testing the deep learning models.

#### The second type of dataset for the conventional machine learning

As conducted in the study of Wolf et al. [20], two new time series were derived from the mean time series for each joint angle: The first gradient time series and the difference from the normative time series. The first gradient “*V*” of the mean time series “*U*” was calculated according to Eq. (1), where “*k*” is the data point index within a gait cycle. A discrete derivation of the mean time series should be done since the time series are in a discrete domain.

1 $$V\left[k\right]=\frac{1}{2}(U\left[k+1\right]-U\left[k-1\right])$$

The difference relative to a reference considered to be normal ${U}_{norm}$, namely difference from normative “$DN"$, was calculated according to Eq. (2):

2 $$DN\left[k\right]=\left|U\left[k\right]-{U}_{norm}\left[k\right]\right|$$

For each joint angle, reference normal time series ${U}_{norm}$ was calculated by averaging the corresponding time series across all TD subjects.

**Fig. 2:**
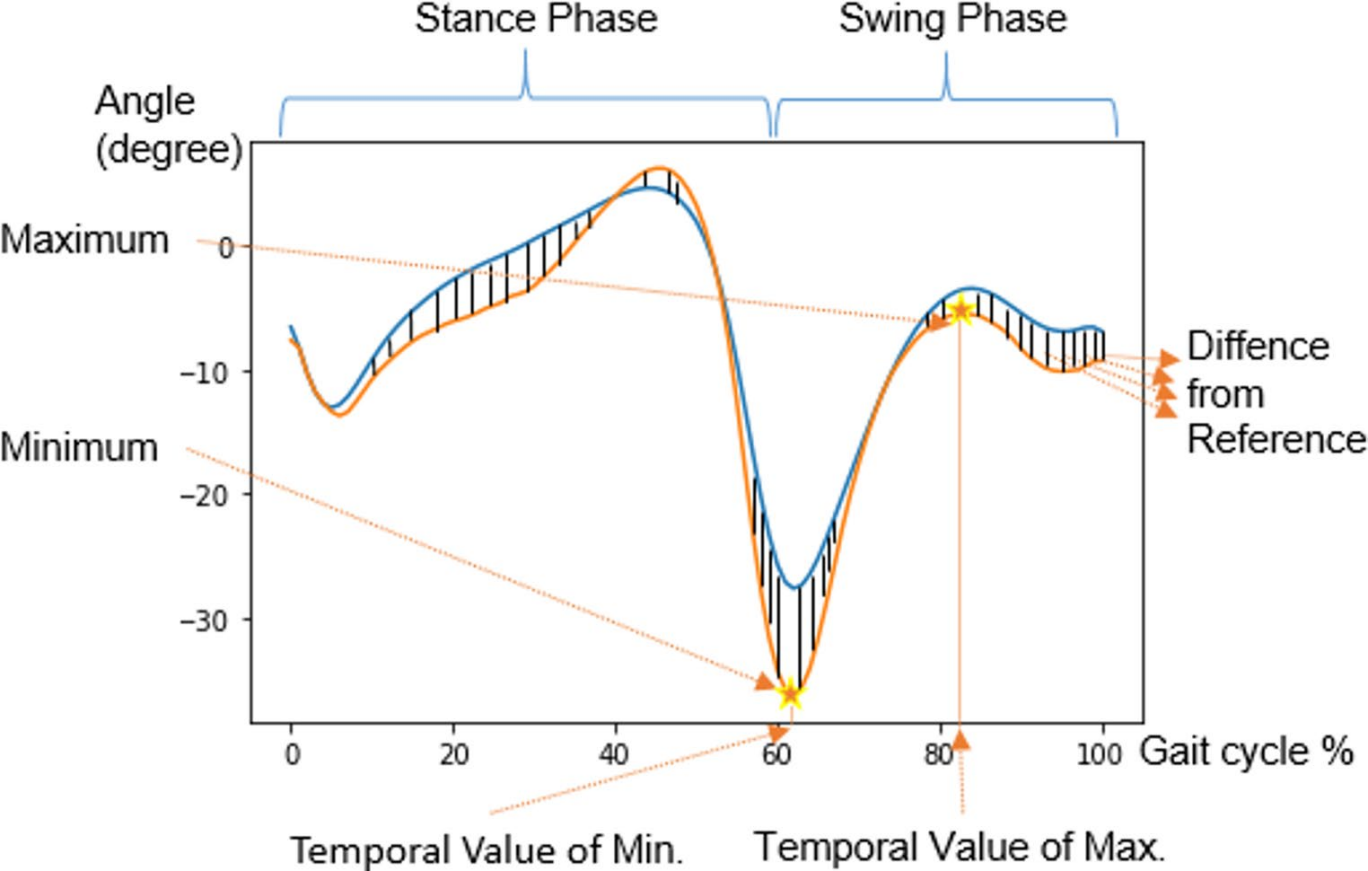
Computed features of an example time series. The orange line shows the ankle dorsi plantar flexion angle of the subject. The blue line shows the reference normal time series for the ankle dorsi plantar flexion

For each joint angle, the computed scalar features from both derived and original time series were as follows: Minimum and maximum values and their timings (i.e. temporal position in the gait cycle; x-axis in Fig. 2). For the standard deviation time series, only the maximum value and its timing were considered as features since it makes sense that strongly varying gait of subjects with CP may cause more differences in the gait patterns among the strides. For the first gradient time series, the average difference from normative data was additionally considered because the difference from the normal gait pattern may contain meaningful information.

**Fig. 3:**
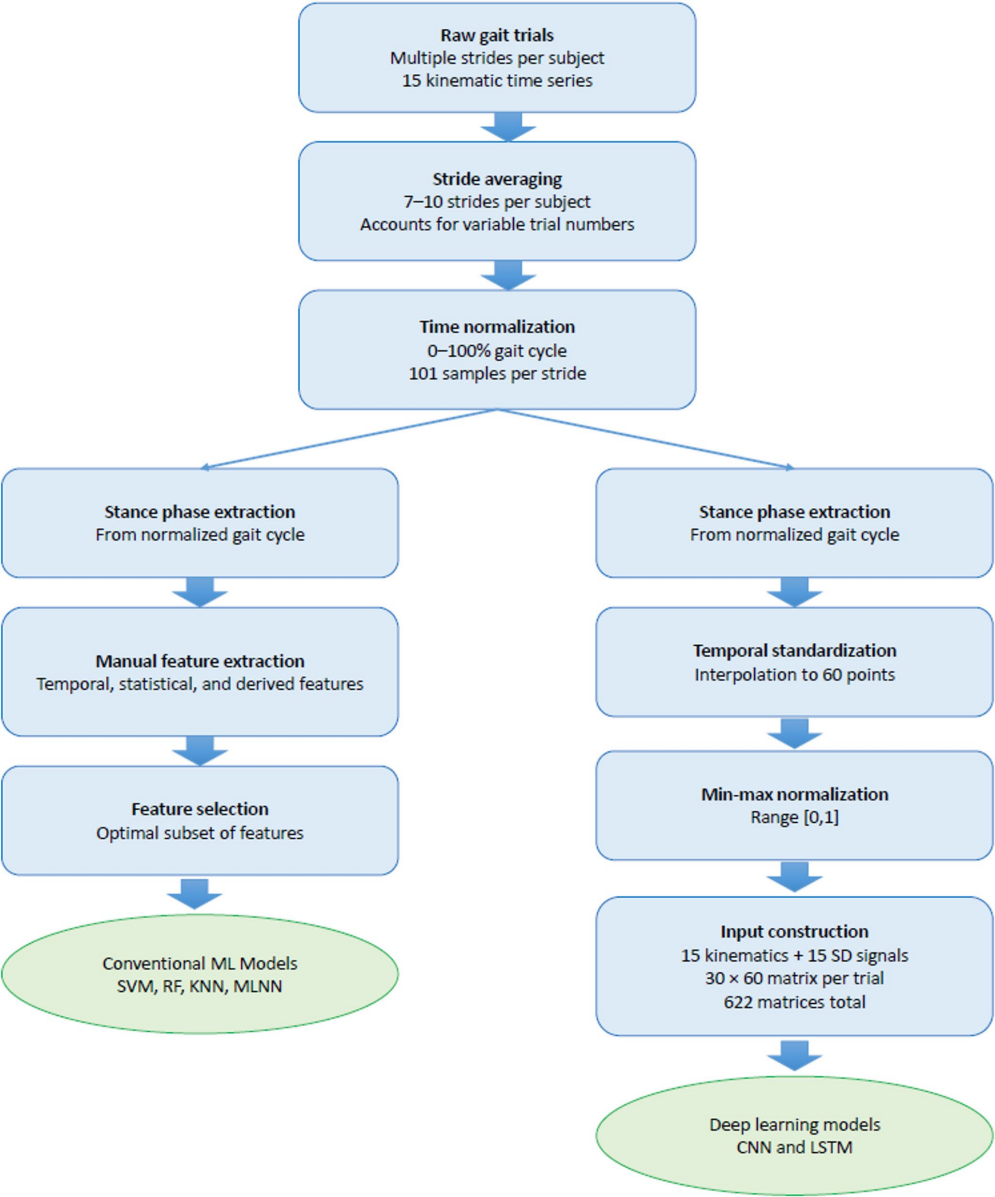
The flowchart for preprocessing of the data and constructing two input datasets

Figure 3 provides a schematic overview of the complete data transformation pipeline, explicitly illustrating stride averaging, time normalization, stance-phase processing, feature extraction, and the construction of inputs for the conventional and deep learning models.

### Machine Learning Methodology

Two deep learning models, namely one-dimensional convolutional neural network (1D-CNN) and long short term memory network (LSTM), and four conventional ML models, namely ridge regression (R-Regr), k-nearest neighbor (KNN), random forest (RF), and multilayer neural network (MLNN) were developed and compared.

#### Hyper-parameter Tuning

Based on the manual preliminary trials conducted on the development set, the hyper-parameters of the ML algorithms were carefully selected to ensure their suitability for our specific case of predicting joint moments.

Hyper-parameters were explored within predefined ranges during development.

For R-Regr, the regularization strength was varied between 0.1 and 10 using singular value decomposition as the solver.

For KNN, the number of neighbours (1–10), leaf size (1–200), distance metrics (Euclidean, Manhattan, Chebyshev, Minkowski), weighting schemes (uniform, distance), and search algorithms (k-d tree, brute force) were evaluated.

For RF, the number of trees (200–1000), maximum depth (10–110), minimum samples per split (2–10), minimum samples per leaf (1–4), feature subsampling strategies (√x, log₂(x), x being the total number of features), split criteria (squared error, Poisson deviance), and bootstrapping options were explored.

For the deep learning models (CNN and LSTM), architectural depth (5–10 dense layers), neuron counts (100–10,000), convolutional filters (128–2048), kernel sizes (3–30), LSTM units (25–100), dropout ratios (0–0.1), ReLU activations for hidden layers, and linear output activations were systematically varied.

The empirically determined optimal structure was subsequently adopted as the MLNN by removing the feature extracting LSTM or CNN layers and by using the abovementioned manually extracted features as input.

The final selected structures and hyper-parameter values for all models are summarized in Table 1.

**Table 1:** Structure and hyper-parameters of the machine learning models

Model	Structure and hyper-parameters of the models
LSTM	One LSTM layer with 50 units, followed by ten fully connected layers with 10,000, 8000, 6000, 4000, 3000, 2000, 1000, 500, 250, and 100 neurons, respectively. A dropout rate of 10% was applied after each layer. Rectified Linear Unit (ReLU) activation was used in the hidden layers, and a linear activation function was used in the output layer.
1D-CNN	Five one-dimensional convolutional layers with 128, 128, 512, 1024, and 2048 filters, and kernel sizes of 30, 15, 10, 5, and 3, respectively. These were followed by ten densely connected layers with 10,000, 8000, 6000, 4000, 3000, 2000, 1000, 500, 250, and 100 neurons. A dropout rate of 10% was applied after each layer. ReLU activation was used in the hidden layers, and a linear activation function was used in the output layer.
R-Regr	Ridge regression with singular value decomposition (SVD) as the solver. The regularization strength was set to 1.0.
KNN	Number of neighbours set to 9. Distance-weighted voting was applied using the inverse of the distances to the query point. Leaf size was set to 49, and the k-d tree algorithm was used for optimization.
RF	Random forest with 366 trees. The minimum number of samples required to split an internal node was set to 5, and the minimum number of samples required at a leaf node was set to 4. The maximum tree depth was limited to 50. Poisson deviance reduction was used as the split criterion. The maximum number of features considered at each split was set to log₂(*x*), where *x* is the total number of features. Bootstrapping was enabled.
MLNN	Ten densely connected layers with 10,000, 8000, 6000, 4000, 3000, 2000, 1000, 500, 250, and 100 neurons, respectively. A dropout rate of 10% was applied after each layer. ReLU activation was used in the hidden layers, and a linear activation function was used in the output layer.

#### Model Development

In the deep learning models, distinct feature extraction from the time series rows was achieved by employing 1D convolution in the CNN model, while the LSTM network utilized long short-term memory cells for the same purpose. The 1D convolution layers were responsible for extracting features from the time series data (specifically joint angles in this study) individually, considering various temporal ranges. On the other hand, LSTM cells extract features based on sequential dependencies of each time point to the previous ones. Both of these deep learning layers sustain valuable information for predicting another time series’ data (in our case joint moments) [21–23]. In the conventional ML models, the manually extracted features were used as inputs to the high degree regression algorithms being trained within the MLNN and R-Regr models and to the ensemble output (in our case each time point of the joint moment) estimation algorithms within the KNN and RF.

**Fig. 4:**
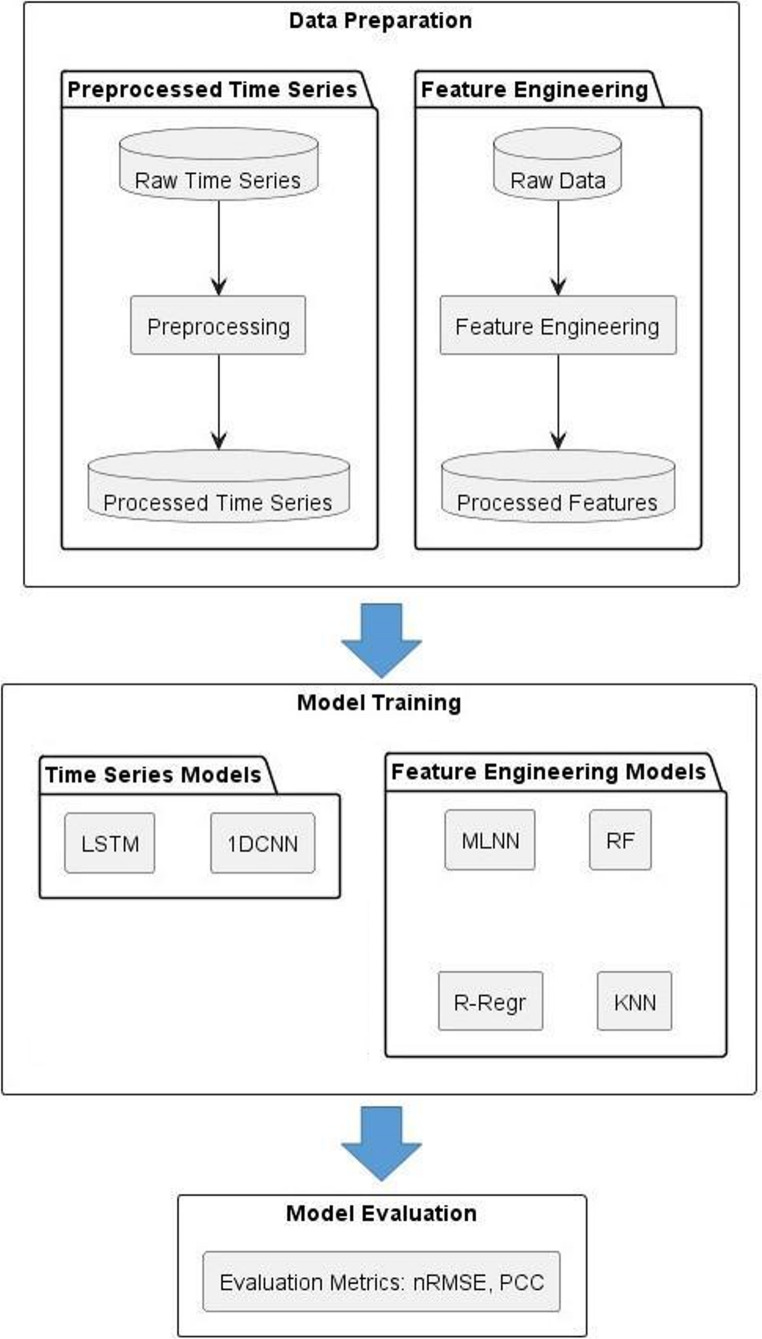
Data processing and machine learning pipeline. MLNN: Multilayer neural network, RF: Random forest, R-Regr: Ridge regression, KNN: K-nearest neighbour, LSTM: Long short term memory neural network, 1DCNN: One dimensional convolutional neural network, nRMSE: normalized root mean square error, PCC: Pearson correlation coefficient

To assess the model’s performance and ensure robustness, a 10-fold cross-validation algorithm was employed. The dataset was divided into 10 equal parts, with nine parts used for training and one part for testing in each fold. Range normalization was applied separately to the training and testing sets to prevent any information leakage between them. Each subject was included in only one of the ten subsets to avoid over-fitting the model to specific walking patterns. The data processing and ML pipelines used in the study were given in Fig. 4.

### Evaluation Metrics and Statistical Analysis

The joint moment time series predictions were evaluated using two well-accepted metrics: the normalized root mean square error (nRMSE (%)) and the Pearson correlation coefficient (PCC). These metrics are commonly used to assess the accuracy of joint moment predictions made by ML algorithms [16, 17, 24, 25].

The nRMSE (%), calculated based on Eq. (3), provides the normalized and time point-wise magnitude difference between the predicted and experimental joint moment time series. To obtain nRMSE, the root mean square error (RMSE) value is divided by the mean range of the experimental joint moment (*µRoM*) across all subjects in the same group. In the equation, ${JM}_{P}$ and ${JM}_{E}$ refer to the predicted and experimental joint moments, respectively, with subindices *P* and *E* indicating predicted and experimental quantities.

3 $$nRMSE=\sqrt{\frac{\sum_n{({JM}_P-{JM}_E)}^2}n}/\mu RoM$$

The PCC calculates the pattern similarity between the experimental and predicted joint moments [26], in which cross-covariance ($cov(E,P)$) of them and variance of each of them (${\sigma}_{E},{\sigma}_{P}$) respectively, were used (Eq. (4)).

4 $$PCC=\frac{cov(E,P)}{{\sigma}_{E}{\sigma}_{P}}$$

To prevent significant skewness in the distribution of the PCC values, we utilized Fisher’s Z transformation. After applying this transformation, we computed the mean using the resulting Z values. Finally, we reversed the mean back to the original PCC scale, following the approach described in the reference [27].

To assess the normality of the data for each joint moment and model, we first performed the Kolmogorov-Smirnov (KS) test. This test compares the sample data to a normal distribution with the same mean and standard deviation. The KS test was applied to the prediction errors (nRMSE or PCC) of each model (CNN, LSTM, RF, MLNN, kNN, R-Regr) across four joint moments: hip flexion-extension, hip adduction-abduction, knee flexion-extension, and ankle dorsiplantar flexion. For each joint moment and model, the null hypothesis of the KS test was that the data follows a normal distribution. If the *p*-value from the KS test was greater than 0.05, we considered the data to be normally distributed; otherwise, we concluded that the data did not follow a normal distribution.

According to the results of the normality tests, the data for all models and joint moments did not follow a normal distribution. Therefore, we applied the Mann-Whitney U test, a non-parametric test, to compare the performance of each deep learning model (CNN and LSTM) with each conventional model (RF, MLNN, kNN, R-Regr) for each joint moment. The Mann-Whitney U test is used to assess whether there are significant differences between the two independent groups, without assuming normality in the data. A significance level of 0.05 was set for all comparisons, and Bonferroni correction was applied to adjust for multiple tests across the joint moments. The test results allowed us to determine whether the deep learning models provided significantly different predictions from the conventional models for each joint moment during gait analysis.

## Results

**Fig. 5:**
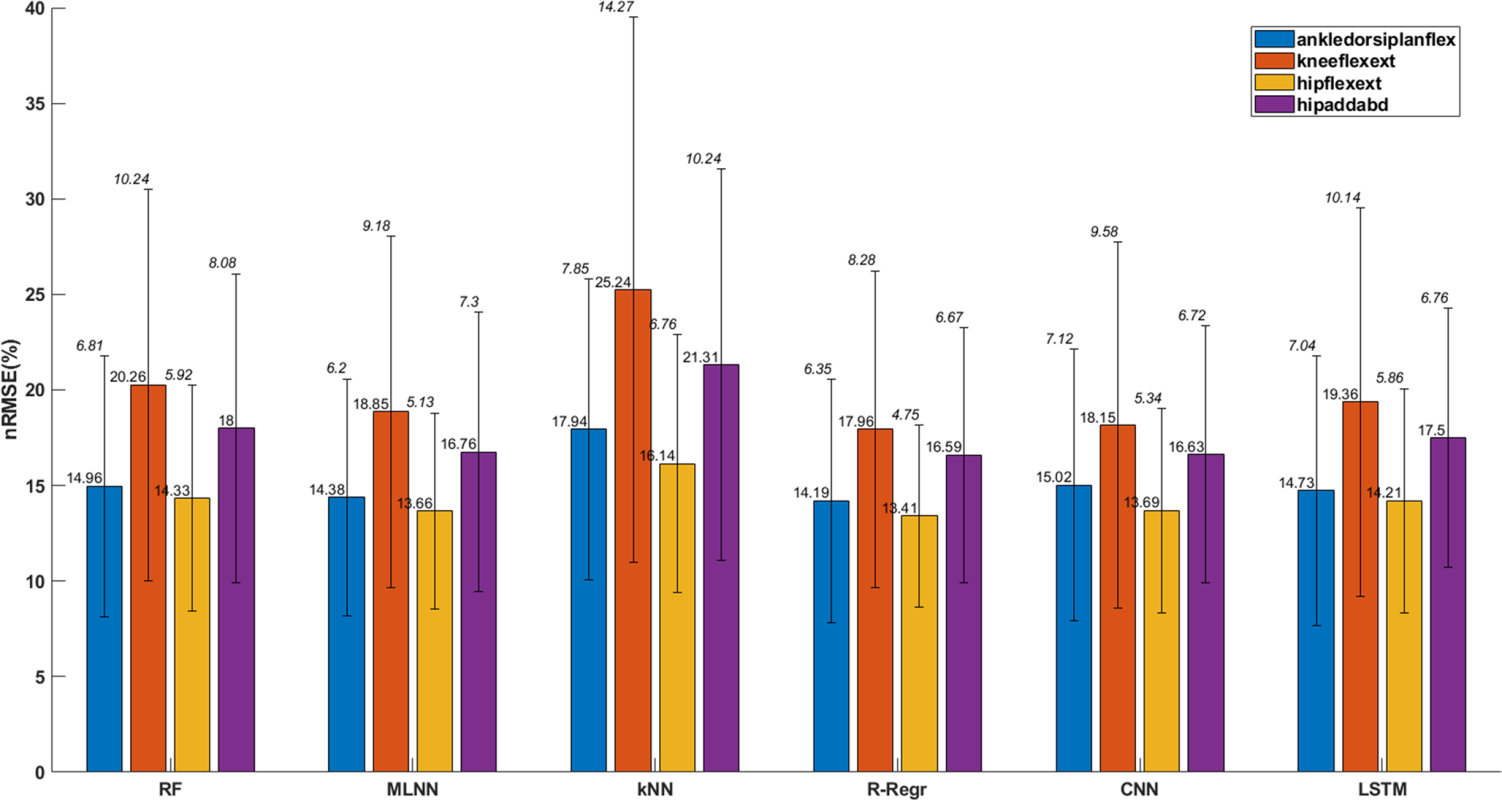
Normalized root mean square error (nRMSE) scores for joint moment predictions of CP subjects. Hip abd/add: hip adduction abduction, hip flex/ext: hip flexion extension, knee flex/ext: knee flexion extension, Dorsi/plant flex: dorsi plantar flexion

The nRMSE values for the models predicting joint moments during gait are presented in Fig. 5. For the RF model, the mean nRMSE ranged from 14.33% for hip flexion-extension to 20.26% for knee flexion-extension. The corresponding standard deviation (SD) values were 5.92% for hip flexion-extension and 10.24% for knee flexion-extension. The MLNN showed mean nRMSE values ranging from 13.66% for hip flexion-extension to 18.85% for knee flexion-extension, with SD values of 5.13% and 9.18%, respectively. For the kNN model, the mean nRMSE values were higher, with the largest error of 25.24% for knee flexion-extension and the lowest error of 16.14% for hip flexion-extension. The SD values for kNN ranged from 6.76% for hip flexion-extension to 14.27% for knee flexion-extension. The R-Regr model demonstrated mean nRMSE values between 13.41% for hip flexion-extension and 17.96% for knee flexion-extension, with SDs ranging from 4.75% to 8.28%.

Deep learning models, including CNN and LSTM, exhibited competitive performance. CNN achieved a mean nRMSE of 13.69% for hip flexion-extension, with an SD of 5.34%, and its highest mean error of 18.15% for knee flexion-extension, with an SD of 9.58%. The LSTM model achieved mean nRMSE values ranging from 14.21% for hip flexion-extension (SD = 5.86%) to 19.36% for knee flexion-extension (SD = 10.14%). Overall, CNN demonstrated relatively consistent performance with smaller variations in SD across joints, while LSTM showed slightly larger variations in error and SD across the different joint moments.

**Fig. 6:**
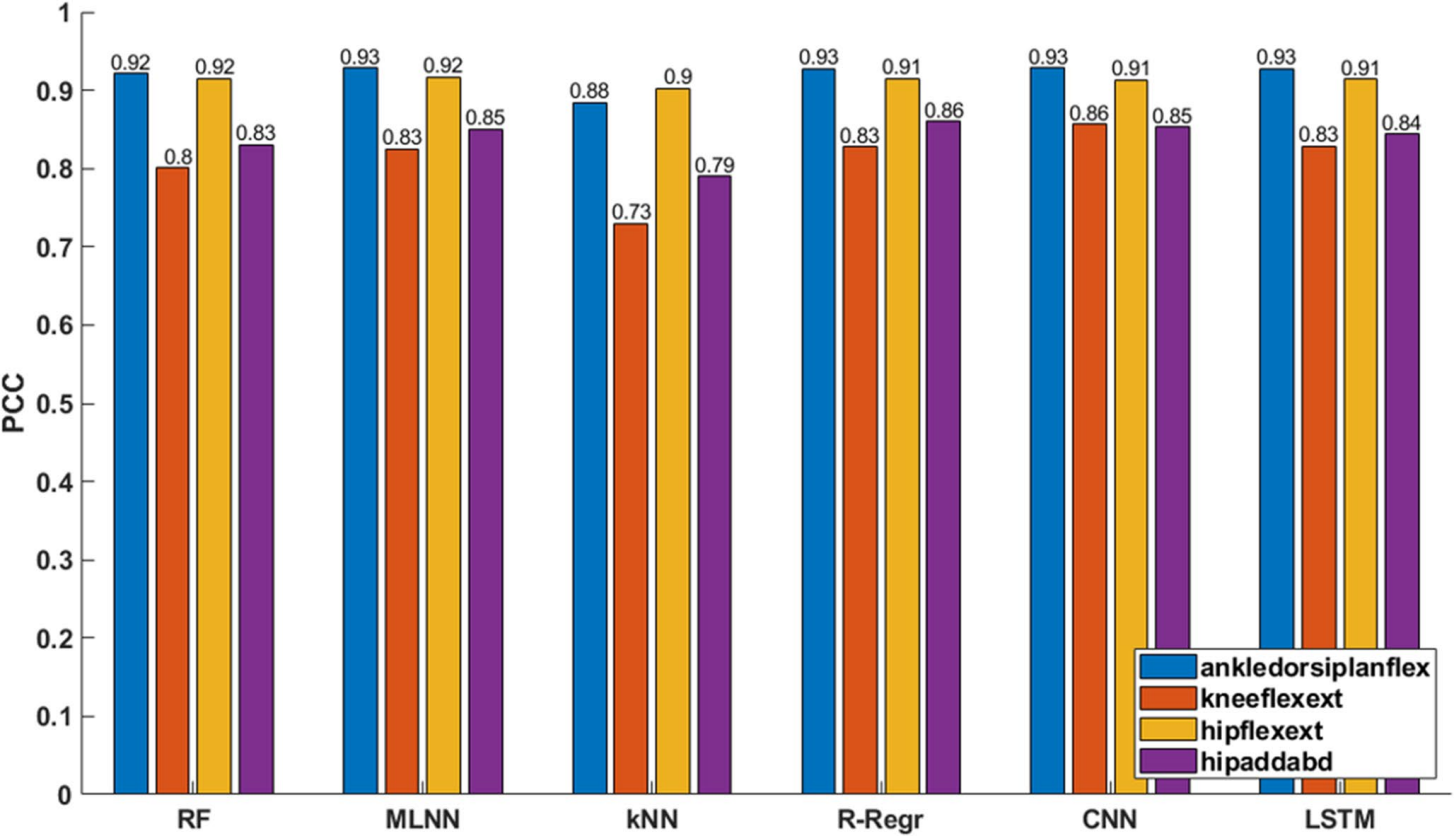
Pearson correlation coefficient (PCC) scores for joint moment predictions of CP subjects. Hip abd/add: hip adduction abduction, hip flex/ext: hip flexion extension, knee flex/ext: knee flexion extension, ankle dorsi/plant flex: dorsi plantar flexion

The PCC values for the models are displayed in Fig. 6. RF achieved consistently high PCC values above 0.80 for all joint moments, with a minimum for knee flexion-extension and with peaks of 0.92 for hip flexion-extension and ankle dorsiplantar flexion. MLNN demonstrated comparable results, achieving PCC values up to 0.93 for ankle dorsiplantar flexion ranging from 0.83 for knee flexion-extension. The kNN model showed lower PCC values compared to RF and MLNN, with its lowest value of 0.73 observed for knee flexion-extension and with a maximum of 0.90 for hip flexion-extension. R-Regr performed similarly to RF and MLNN, reaching a maximum PCC of 0.93 for ankle dorsiplantar flexion and having a minimum of 0.83 for knee flexion-extension.

Deep learning models also exhibited high PCC values across joints. CNN achieved a maximum PCC of 0.93 for ankle dorsiplantar flexion, with slightly lower values for other joints, such as 0.85 for hip adduction-abduction and 0.86 for knee flexion-extension. The LSTM model demonstrated PCC values ranging from 0.83 for knee flexion-extension to 0.93 for ankle dorsiplantar flexion, indicating high correlation performance across the predicted joint moments. Both CNN and LSTM models showed competitive results in terms of PCC values across all joints.

The tables in Appendix 1 show the statistical significance of the obtained nRMSE and PCC scores between deep learning models and conventional models for each joint moment.

**Fig. 7:**
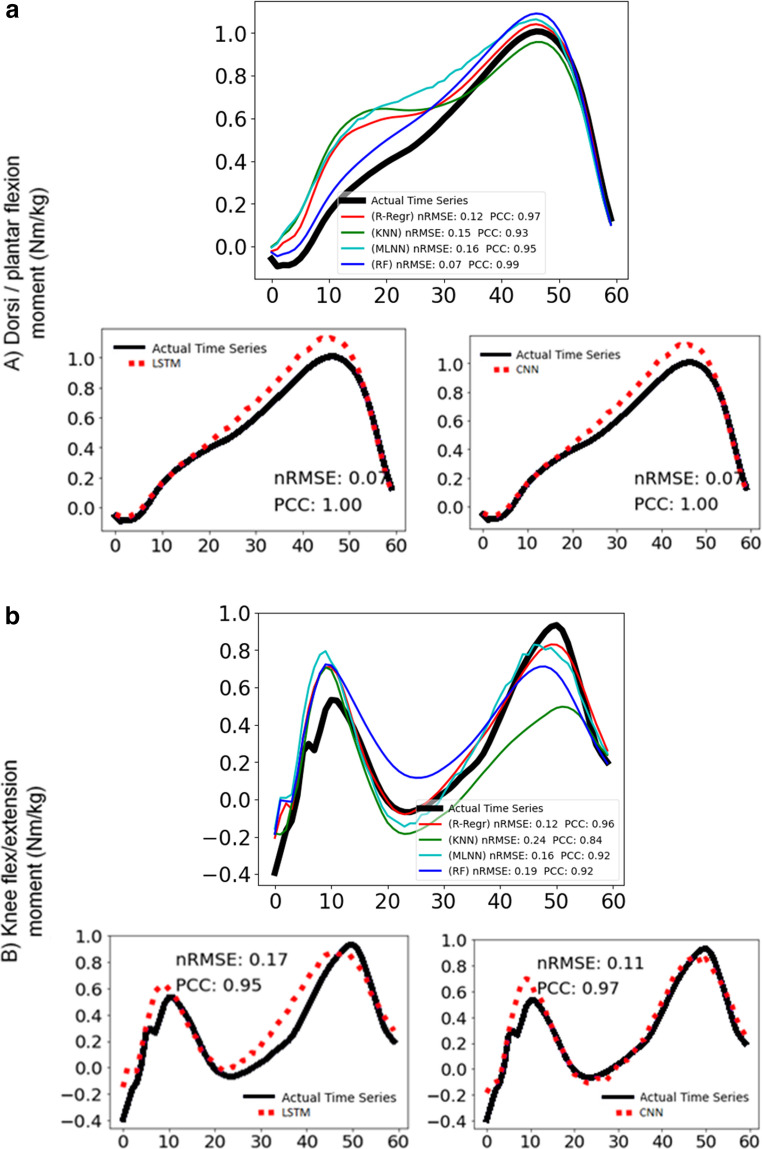

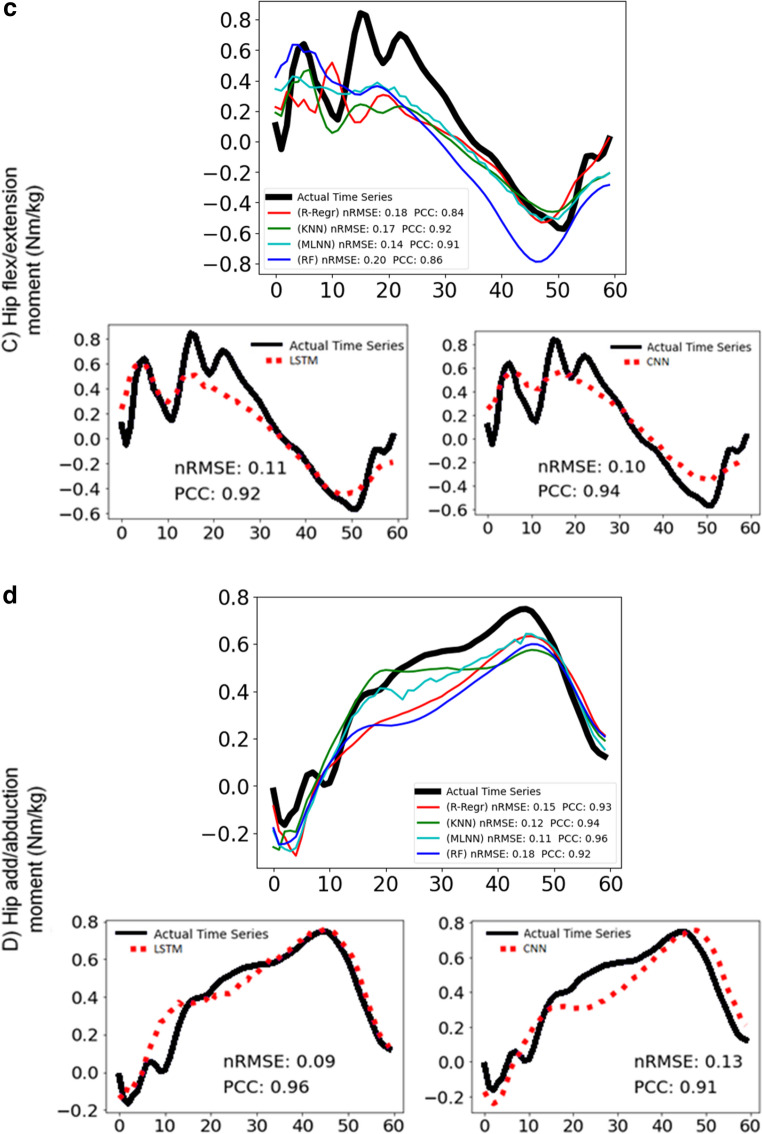
Joint moment predictions with above average success of most of the models for (**a**) ankle dorsi-plantar flexion, **b** knee flexion-extension, **c** hip flexion-extension, **d** hip adduction-abduction with conventional and deep learning models. For each moment: the graph above shows the actual time series (black line) and the predictions of the ridge-regression (red line), k-nearest neighbour (green line), multilayer neural network (cyan line), random forest (blue line) and the graphs below show the actual time series (black line) and the predictions of long-short term memory network (red dotted line) on the left, convolutional neural network (red dotted line) on the right. The nRMSE and PCC scores are noted in the graphs

**Fig. 8:**
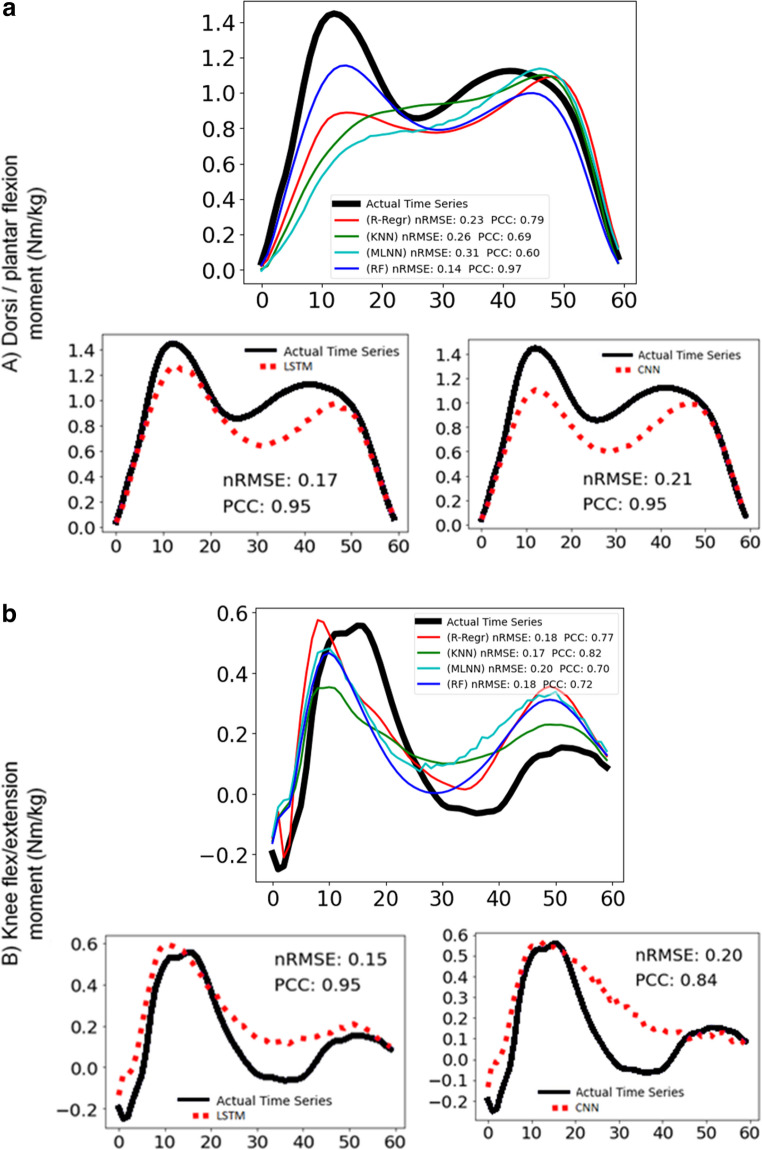

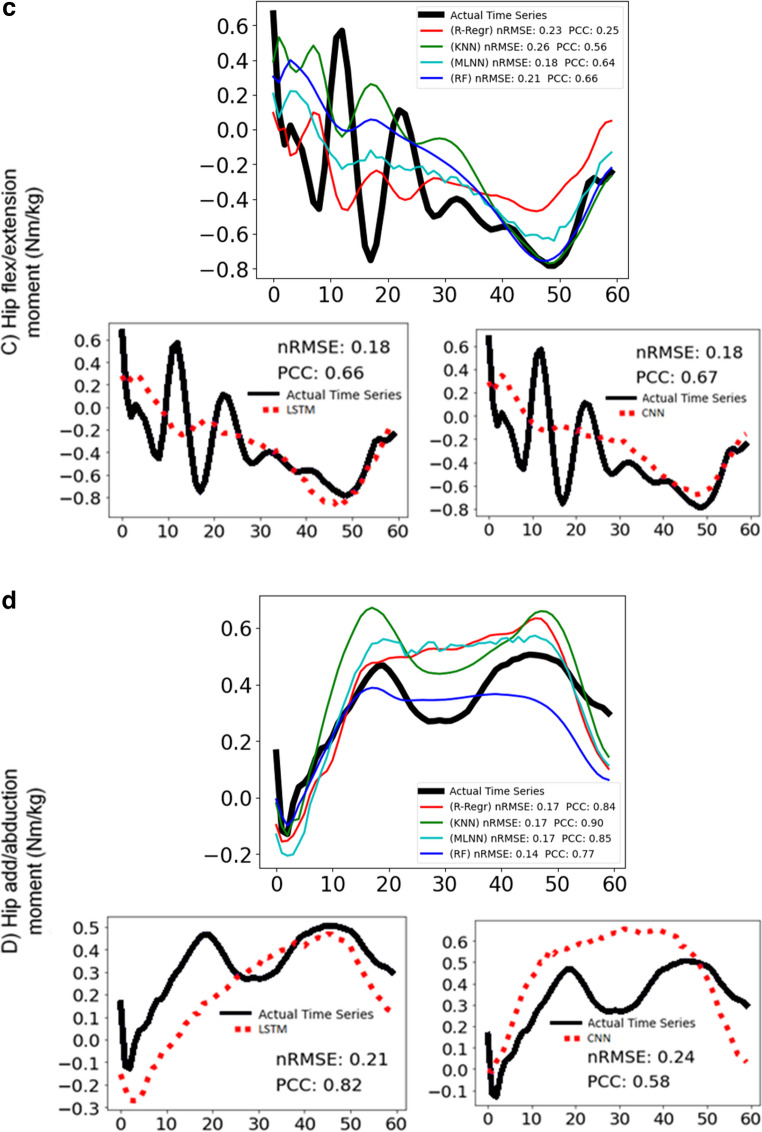
Joint moment predictions with below average success of most of the models for (**a**) dorsi-plantar flexion, **b** knee flexion-extension, **c** hip flexion-extension, **d** hip adduction-abduction with conventional and deep learning models. For each moment: the graph above shows the actual time series (black line) and the predictions of the ridge-regression (red line), k-nearest neighbour (green line), multilayer neural network (cyan line), random forest (blue line) and the graphs below show the actual time series (black line) and the predictions of long-short term memory network (red dotted line) on the left, convolutional neural network (red dotted line) on the right. The nRMSE and PCC scores are noted in the graphs

Figure 7 and 8 display representative examples of predicted and experimental joint moments obtained using both conventional machine learning and deep learning algorithms. These figures are included to help illustrate the models’ ability to predict joint moments with varying patterns. To highlight the models’ performance, Fig. 7 presents predictions that are relatively more accurate, showing lower nRMSE and higher PCC values compared to the average. In contrast, Fig. 8 presents predictions that are less accurate, characterized by higher nRMSE and lower PCC values than the average

## Discussion

Due to the significance of joint moments as crucial assessment parameters in managing CP [1], and considering the recent advancements in ML for predicting these moments [18], we conducted an extensive analysis. Our study utilized both raw-data-based and feature-based data representation methods and involved training six ML algorithms (kNN, MLNN, RF, R-Regr, LSTM, and 1DCNN) spanning both conventional and deep learning approaches. The primary objective was to comparatively predict lower extremity joint moments during gait in individuals with CP using gait kinematics data. To the best of our knowledge, the literature lacks any study that directly compares conventional ML and deep learning approaches for joint moment prediction in CP patients.

We found that most models, except for kNN, demonstrated comparable levels of performance, with relatively low standard deviations. This suggests that reasonably accurate predictions of gait kinetics can be achieved regardless of the specific kinematic data representation method, provided that appropriate ML algorithms are employed during training. Consequently, both manually extracted time-domain features and automatically extracted features within deep learning models appear to provide sufficient information for effectively predicting joint moments in individuals with CP.

It is worth noting that conventional ML algorithms tend to be more straightforward to train when the provided information is relevant and representative. In other words, when the input features accurately capture the essential characteristics of the data, conventional algorithms are typically better equipped to learn and make accurate predictions. Therefore, in this study, careful consideration was given to selecting and constructing input sets that encompassed pertinent and meaningful information from the kinematic data regarding the study of Wolf et al. [20], aiming to enhance the training process and optimize the performance of the ML models.

Deep learning, in contrast, has the ability to automatically create and extract its own features from the input data [18]. It does not rely on handcrafted features like conventional ML approaches. By doing so, deep learning models can explore a much wider solution space, allowing them to capture complex patterns and relationships in the data.

However, deep learning algorithms typically require a larger amount of data to effectively learn these complex representations [28]. With a substantial amount of data available for training, deep learning models can leverage their capacity to learn intricate patterns and achieve remarkable performance on more challenging and complex problems. The abundance of data enables the models to generalize well and make accurate predictions or classifications [29].

Therefore, deep learning works best when there is plenty of high-quality, representative data to learn from. In the practical context of clinical gait analysis for individuals with CP, this includes kinematic recordings that are consistently captured across multiple gait cycles, cover the full range of clinically relevant movement patterns, and reflect variability in walking patterns. When such data are available in adequate quantity, deep models are better able to capture complex, hierarchical relationships in gait dynamics and may outperform conventional ML methods.

The influence of hyper-parameter selection further highlights this interaction between model structure and data characteristics. In RF models, parameters governing tree depth, minimum samples per split, and leaf size appeared to affect the bias–variance trade-off, in that deeper trees with minimal constraints tended to reduce training error while being associated with less stable cross-validation performance, whereas moderate depth and leaf constraints were generally linked to improved generalization across individuals. In KNN models, the number of neighbours seemed to modulate sensitivity to individual gait patterns, with smaller neighbourhoods emphasizing subject-specific variations and larger neighbourhoods leading to more smoothed predictions. For deep learning models, architectural depth, number of filters or units, and dropout ratios were observed to influence performance trends. Shallow or under-parameterized networks were associated with limited capture of temporal structure, while excessively deep or weakly regularized networks showed indications of overfitting, particularly in subjects with atypical gait patterns. Consequently, the final hyper-parameter configurations were selected not as isolated optima, but as balanced solutions that consistently yielded stable performance across cross-validation folds. Taken together, these findings suggest that hyper-parameters are not merely implementation details, but may shape how biomechanical information is represented and generalized by each model.

One of the findings of our study is that various kinematic data representation methods resulted in similar prediction performance. This implies that the decision between manual feature extraction and automatic feature extraction (with their matching deep and conventional ML algorithms) can be made based on practical considerations, primarily considering the availability of data for the specific task of predicting gait kinetics.

The fact that the strongly varying gait deviations in patients with CP, allows us to expect the studied ML algorithms to perform similarly with other patients’ data, having similar or less deviation within their testing samples.

Prior works explored the mapping between kinematic information and kinetic outputs using both manually extracted features and data-driven representations. For example, Wolf et al. demonstrated that automated assessment of clinically meaningful kinematic features could support instrumented gait analysis [20]. In parallel, more recent studies have adopted convolutional and recurrent neural networks to learn representations directly from raw time series, thereby reducing the need for explicit feature engineering [16, 30]. In our study, these two paradigms were evaluated in parallel by pairing conventional ML models with manually extracted kinematic features, and deep learning models with full time series. This design enabled a controlled comparison of how different model families exploited biomechanical information under input representations that are commonly used in gait analysis.

Some limitations of this study should be taken into account. Incorporating additional input data beyond lower limb joint kinematics, such as temporospatial parameters or electromyography signals, could potentially improve model performance. However, since the data representation was kept consistent across models, the comparative evaluation between conventional and deep learning approaches is still expected to yield meaningful and reliable insights. Another limitation is that, despite the relatively large dataset, it includes only patients with spastic diplegia. This limits the generalizability of the findings to other CP subtypes, such as hemiplegia, which may involve different gait characteristics and joint moment profiles.

From a clinical perspective, kinematic-only prediction of joint moments has the potential to complement conventional instrumented gait analysis by reducing dependence on force plates and sophisticated laboratory infrastructure. This could enable more accessible biomechanical assessment in settings where full kinetic measurements are impractical or unavailable, such as outpatient clinics, rehabilitation centers, and longitudinal follow-up studies. Although further validation is required before clinical deployment, these findings suggest that kinematic-driven models could help bridge the gap between laboratory-based gait analysis and real-world clinical assessment.

In conclusion, both conventional and deep learning approaches showed promise for predicting joint moments in patients with CP. By considering factors such as data availability and computational cost, an appropriate ML method can be selected to effectively address gait kinetics prediction in individuals with CP. Clinically, this suggests that meaningful biomechanical information may be obtained even in the absence of full kinetic instrumentation, supporting broader and more flexible use of gait analysis in routine clinical practice.

## Appendix 1

**Table 2:** Mann-Whitney U Test results for PCC between the deep learning and conventional machine learning models

Joint	Model Comparison	*p*-value
**Ankledorsiplanflex**	CNN vs. Ridge	**0.0383**
	CNN vs. kNN	0.1631
	CNN vs. MLNN	0.0713
	CNN vs. RF	**0.0219**
	LSTM vs. Ridge	**0.0415**
	LSTM vs. kNN	0.1729
	LSTM vs. MLNN	0.1004
	LSTM vs. RF	**0.0215**
**Kneeflexext**	CNN vs. Ridge	**0.0014**
	CNN vs. kNN	**0.0006**
	CNN vs. MLNN	**0.0011**
	CNN vs. RF	0.0068
	LSTM vs. Ridge	0.1068
	LSTM vs. kNN	0.0723
	LSTM vs. MLNN	0.0695
	LSTM vs. RF	0.2124
**Hipflexext**	CNN vs. Ridge	0.7949
	CNN vs. kNN	0.5223
	CNN vs. MLNN	0.8569
	CNN vs. RF	0.5328
	LSTM vs. Ridge	0.9039
	LSTM vs. kNN	0.5858
	LSTM vs. MLNN	0.9329
	LSTM vs. RF	0.6211
**Hipaddabd**	CNN vs. Ridge	0.2017
	CNN vs. kNN	0.1506
	CNN vs. MLNN	0.6138
	CNN vs. RF	0.0982
	LSTM vs. Ridge	0.5845
	LSTM vs. kNN	0.4579
	LSTM vs. MLNN	0.8513
	LSTM vs. RF	0.3283

Significant differences were marked bold. Hipaddabd: hip adduction abduction, hipflexext: hip flexion extension, kneeflexext: knee flexion extension, ankledorsiplanflex: ankle dorsi plantar flexion

**Table 3:** Mann-Whitney U Test results for nRMSE between the Deep Learning and Conventional Machine Learning Models. Significant differences were marked bold. Hipaddabd: hip adduction abduction, hipflexext: hip flexion extension, kneeflexext: knee flexion extension, ankledorsiplanflex: ankle dorsi plantar flexion

Joint	Model Comparison	*p*-value
**Ankledorsiplanflex**	CNN vs. Ridge	0.8729
	CNN vs. kNN	0.9029
	CNN vs. MLNN	0.3795
	CNN vs. RF	0.6247
	LSTM vs. Ridge	0.7516
	LSTM vs. kNN	0.5227
	LSTM vs. MLNN	0.1646
	LSTM vs. RF	0.9689
**Kneeflexext**	CNN vs. Ridge	**0.0092**
	CNN vs. kNN	**0.0217**
	CNN vs. MLNN	0.0501
	CNN vs. RF	0.1989
	LSTM vs. Ridge	0.0988
	LSTM vs. kNN	0.2200
	LSTM vs. MLNN	0.2979
	LSTM vs. RF	0.8521
**Hipflexext**	CNN vs. Ridge	0.9875
	CNN vs. kNN	0.9619
	CNN vs. MLNN	0.8917
	CNN vs. RF	0.5078
	LSTM vs. Ridge	0.5111
	LSTM vs. kNN	0.6679
	LSTM vs. MLNN	0.5532
	LSTM vs. RF	0.2297
**Hipaddabd**	CNN vs. Ridge	0.9777
	CNN vs. kNN	0.9890
	CNN vs. MLNN	0.9553
	CNN vs. RF	0.3569
	LSTM vs. Ridge	0.3087
	LSTM vs. kNN	0.3614
	LSTM vs. MLNN	0.4058
	LSTM vs. RF	0.9721
